# Normal saline vs. lactated ringer’s for fluid resuscitation in acute pancreatitis: a systematic review and meta-analysis

**DOI:** 10.3389/fmed.2026.1792711

**Published:** 2026-05-08

**Authors:** Weijun Jiang, Jingwen Peng, Qiuyue Wu, Shengjie Wang, Peng Zheng, Xinyi Xia

**Affiliations:** 1 State Key Laboratory of Coordination Chemistry, School of Chemistry and Chemical Engineering, Nanjing University, Nanjing, Jiangsu, China; 2 State Key Laboratory of Analytical Chemistry for Life Science, Institute of Laboratory Medicine, Jinling Hospital, Affiliated Hospital of Medical School, Nanjing University, Nanjing, Jiangsu, China; 3 Department of Critical Care Medicine, Center of Severe Acute Pancreatitis (CSAP), Jinling Hospital, Affiliated Hospital of Medicine School, Nanjing University, Nanjing, Jiangsu, China; 4 Department of Basic Medicine, Kangda College of Nanjing Medical University, Lianyungang, Jiangsu, China; 5 Lianyungang Center for Medical Education and Innovation Research of Nanjing Medical University, Lianyungang, Jiangsu, China; 6 Jinling Hospital, The First School of Clinical Medicine, Southern Medical University, Nanjing, Jiangsu, China

**Keywords:** acute pancreatitis, fluid resuscitation, lactated ringer, meta-analysis, normal saline

## Abstract

**Background:**

Intravenous fluid resuscitation is fundamental in acute pancreatitis (AP), but optimal crystalloid selection remains debated. This meta-analysis compares lactated ringer’s (LR) and normal saline (NS) for outcomes in AP.

**Methods:**

We conducted the systematic review and meta-analysis in accordance with the PRISMA 2020 guidelines. We systematically searched PubMed, Web of Science, Embase, CNKI, and WanFang (Until July 2025) for studies comparing LR and NS in AP. Primary outcomes included mortality, systemic inflammatory response syndrome (SIRS), ICU transfer, pancreatic necrosis, organ failure, and hospital length of stay (LOS). Random- or fixed-effects models were used based on heterogeneity (*I*^2^).

**Results:**

Overall mortality did not differ between LR and NS (OR = 1.020, 95% CI: 0.865–1.359, *p* = 0.895). No mortality benefit was observed for LR in any severity subgroup, including severe acute pancreatitis (SAP) (OR = 0.931, 95% CI: 0.157–5.505, *p* = 0.937). LR significantly shortened LOS in RCTs (SMD = −0.307, 95% CI: −0.511 to −0.102, *p* = 0.003) and small-sample studies (<100 cases; SMD = −0.271, 95% CI: −0.519 to −0.022, *p* = 0.033), but the overall pooled analysis showed no significant difference (SMD = −0.121, *p* = 0.201). Notably, SAP patients receiving LR had a longer LOS (SMD = 0.368, 95% CI: 0.072–0.664, *p* = 0.015). No significant differences were observed for SIRS, ICU transfer, pancreatic necrosis, or organ failure, though organ failure showed a non-significant trend favoring LR (OR = 0.563, *p* = 0.063). High heterogeneity existed in non-RCT and large-sample (>100 cases) subgroups. Trial sequential analysis confirmed true negative findings for mortality, ICU transfer and SIRS, but indicated insufficient power for pancreatic necrosis, organ failure and LOS.

**Conclusion:**

LR and NS demonstrate comparable mortality and safety profiles, with no significant difference in overall LOS. Although LR may shorten LOS in certain subgroups, its association with prolonged LOS in patients with SAP requires further validation. Therefore, based on current evidence, no preference can be recommended for either fluid, and larger standardized trials are warranted.

**Systematic review registration:**

CRD420251112118.

## Introduction

The incidence of acute pancreatitis (AP) occurs between 4.9 and 73.4 per 100,000 people per year worldwide (1, 2). It is the second most common cause of hospitalization, with a very high mortality rate despite urgent and necessary treatment. The mainstay of early management in AP is intravenous fluid resuscitation, which counteracts hypovolemia and its consequence, organ hypoperfusion (3–5). There is still limited consensus, however, on the specifics of fluid resuscitation regimens, particularly the choice of fluid type, despite some guidelines advocating an aggressive approach.

Recent studies have increasingly supported the use of moderate rather than aggressive fluid resuscitation volumes in AP. However, the optimal type of crystalloid solution remains uncertain (3). This question is especially relevant in severe acute pancreatitis (SAP), where mortality rates can reach approximately 20% (6). A recent randomized controlled trial (RCT) showed that lactated ringer’s solution (LR), comparing to normal saline (NS), may lower the need for intensive care and reduce hospital length of stay in AP patients (7). This finding has been further supported in recent years by several systematic reviews and meta-analyses comparing LR and NS (1, 8, 9). Nevertheless, limitations such as small sample sizes, low incidence of moderate-to-severe cases, and scarce reporting of systemic complications hinder meaningful conclusions regarding the benefits of LR.

Therefore, this meta-analysis aims to synthesize current evidence to evaluate the efficacy and safety of LR versus NS in AP treatment. Compared with prior meta-analyses, our study includes the largest sample to date (*N* = 3,090), incorporates trial sequential analysis (TSA) to distinguish true negative from false negative findings, and performs severity-stratified subgroup analyses to address previous inconsistencies regarding SAP outcomes.

## Materials and methods

### Search strategy

The methodology for this systematic review and meta-analysis was guided by the PRISMA 2020 statement. We systematically searched PubMed, Web of Science, Embase, CNKI, and WanFang databases for relevant records up to July 2025. The search employed the following combination of terms: (“Lactated Ringer’s” OR “Ringer’s lactate”) AND (“normal saline” OR “saline”) AND (“acute pancreatitis”) AND (“fluid resuscitation”).

### Study selection

**Figure 1:**
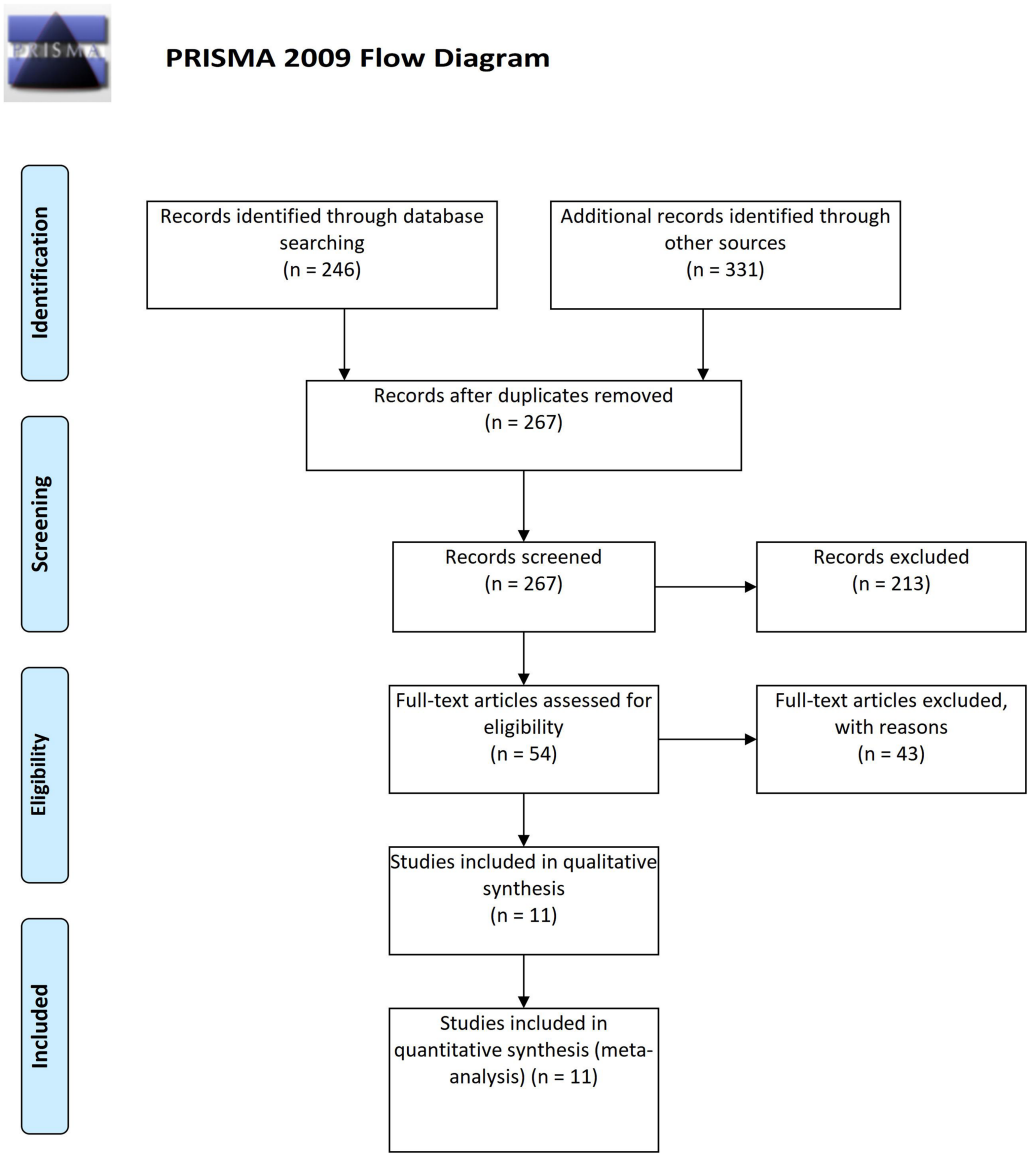
Flow diagram depicting the search and selection strategy (28).

The required data were retrieved from each of the eligible published articles (Figure 1). Inclusion criteria: RCTs or observational studies comparing LR and NS in AP. Reporting ≥1 outcome: mortality, systemic inflammatory response syndrome (SIRS), transfer to ICU, pancreatic necrosis, organ failure, or length of stay (LOS). Exclusion criteria: Non-human studies, reviews, or insufficient data. As a systematic review and meta-analysis of previously published data, this study did not require ethical approval or informed consent.

### Data extraction and quality assessment

Disease severity encompassed mild acute pancreatitis (AP), moderately severe acute pancreatitis (MAP), and severe acute pancreatitis (SAP). Two authors independently extracted: first author, year, country, race, design, case/control sizes, severity (AP/MAP/SAP), and outcomes. Disagreements were resolved by review and discussion. Information pertaining to the enrolled studies is listed in Table 1.

**Table 1:** Baseline characteristics of included studies (total *N* = 3,090 patients).

Author(year)	Country	Race	Study design	Cases/Controls	Severity	Case size	Death	Length of stay	Systemic inflammatory response syndrome (SIRS)	ICU transfer	Pancreatic necrosis	Organ failure
							Case/Control	Case/Control	Case/Control	Case/Control	Case/Control	Case/Control
Wu et al. (14)	USA	Mix	RCT	19/21	AP	<100	0/0	4.6397 ± 2.4029/6.2186 ± 2.3851	6/4	1/3	0/1	1/4
Lipinski et al. (15)	Poland	Caucasian	Retrospective study	40/63	SAP	>100	5/3				10/12	
Aboelsoud et al. (16)	USA	Mix	Retrospective study	68/130	SAP	>100	4/21	6.2 ± 6.9/4.2 ± 4.49				
de-Madaria et al. (17)	Spain	Caucasian	RCT	19/21	AP	<100	0/1	9.2882 ± 5.767/10.0779 ± 8.7454	9/14	0/1	4/10	0/1
Choosakul et al. (18)	Thailand	Asian	RCT	23/24	MAP	<100	0/1	6.3585 ± 3.9509/6.2163 ± 3.9403	8/10		0/2	
Kayhan et al. (19)	Turkey	Caucasian	Prospective study	67/65	MAP	>100		3 ± 1.5153/3.353 ± 2.2745			2/5	6/13
Lee et al. (20)	USA	Mix	RCT	61/60	MAP	>100	0/0	3.8179 ± 2.9612/5.0239 ± 3.3422	17/14	6/15		7/9
Karki et al. (21)	Nepal	Asian	RCT	26/25	MAP	<100	0/1	5.15 ± 0.09/6.20 ± 2.5	0/3			
Antoniak et al. (22)	USA	Mix	Retrospective study	2027/18022	AP	>100	40/310	4.0851 ± 2.8932/4.085 ± 2.8913	386/3808	15/96		
Farrell et al. (7)	USA	Mix	RCT	38/38	AP	<100	0/0	77.0886 ± 61.6335/84.6167 ± 42.373		4/2	2/1	
Lee et al. (12)	USA	Mix	Prospective study	328/364	AP	>100			122/97	24/26		

### Statistical analysis

Outcomes were pooled and expressed as odds ratios (ORs) or standardized mean differences (SMDs) with corresponding 95% confidence intervals (CIs). Study heterogeneity was assessed using the *I*^2^ statistic and the *p*-value of the heterogeneity test. An *I*^2^ < 50% and a *p*-value > 0.10 indicated acceptable homogeneity, in which case a fixed-effects model (Mantel–Haenszel method) was applied to calculate the summary OR. Conversely, an *I*^2^ ≥ 50% or a p-value ≤ 0.10 indicated significant heterogeneity. In such cases, provided the studies still met our inclusion criteria, a random-effects model (DerSimonian-Laird method) was used to estimate the summary OR. Subgroup analyses were performed based on race, study design (RCT vs. non-RCT), sample size (<100 vs. ≥100), and disease severity. Sensitivity analyses were conducted by sequentially removing individual studies, and publication bias was assessed using Egger’s test. All statistical analyses were performed with STATA software, version 17.0 (Stata Corporation, College Station, TX, United States).

## Results

### Study characteristics

Eleven studies (2011–2023; *N* = 3,090 patients) were included (Table 1). Six were RCTs; five were observational studies (three prospective, two retrospective). Study populations comprised diverse ethnicities: multi-ethnic (n = 5), Caucasian (n = 3), and Asian (n = 3).

### Primary outcomes

**Figure 2:**
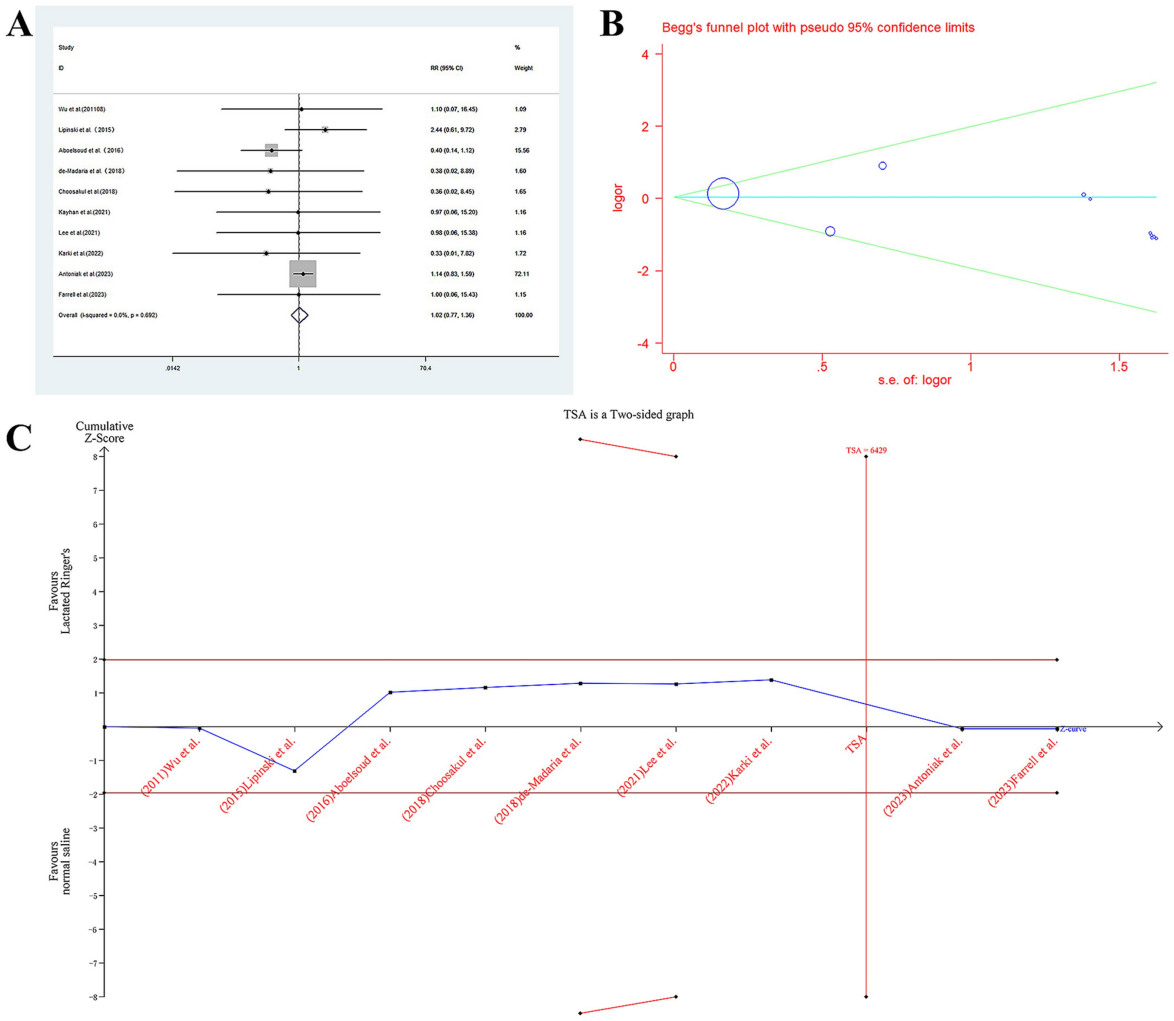
The relationship between LR and NS and the rate of mortality. **(A)** Forest plot for overall analysis **(B)** The funnel plot for the association between LR and NS and the rate of mortality **(C)** Trial sequential analysis of LR and NS and the rate of mortality.

Overall mortality did not differ significantly between the LR and NS groups (OR = 1.020, 95% CI: 0.865–1.359; *p* = 0.895; *I*^2^ = 0.0%). This finding remained consistent across all prespecified subgroup analyses stratified by race (Mix: OR = 1.013, *p* = 0.935; Caucasian: OR = 1.542, *p* = 0.434; Asian: OR = 0.347, *p* = 0.352), study design (RCT: OR = 0.630, *p* = 0.432; non-RCT: OR = 1.057, *p* = 0.725), case size (<100: OR = 0.573, *p* = 0.393; >100: OR = 1.054, *p* = 0.725), and disease severity (AP: OR = 1.128, *p* = 0.470; SAP: OR = 0.931, *p* = 0.937; MAP: OR = 0.597, *p* = 0.485), with no subgroup achieving statistical significance (all *p* > 0.05). The specific results can be seen in Figure 2 and Table 2.

**Table 2:** Mortality outcomes in acute pancreatitis: meta-analysis of LR vs. NS.

	OR (95% CI)	*P*	*P_h_*	*I*^2^%	*P*_b_
Total	1.020(0.865–1.359)	0.895	0.692	0.0%	0.236
Race					
Mix	1.013(0.749–1.370)	0.935	0.452	0.0%	
Caucasian	1.542(0.521–4.559)	0.434	0.525	0.0%	
Asian	0.347(0.037–3.226)	0.352	0.972	0.0%	
Design					
RCT	0.630(0.199–1.997)	0.432	0.980	0.0%	
NO RCT	1.057(0.777–1.437)	0.725	0.156	42.6%	
Case size					
<100	0.573(0.159–2.059)	0.393	0.958	0.0%	
>100	1.054(0.785–1.417)	0.725	0.280	21.2%	
Severity					
AP	1.128(0.814–1.564)	0.470	0.925	0.0%	
SAP	0.931(0.157–5.505)	0.937	0.039	76.6%	
MAP	0.597(0.140–2.542)	0.485	0.922	0.0%	

CI, confidence interval; LR, lactated ringer’s; NS, normal saline; OR, odds ratio; RCT, randomized controlled trial; SAP, severe acute pancreatitis. Fixed-effects model used for overall analysis (*I*^2^ = 0.0%). Subgroups stratified by race, study design, case size, and disease severity. All comparisons non-significant (*P* > 0.05).

No significant intergroup difference was detected in SIRS between LR and NS groups (OR = 0.981, 95% CI: 0.723–1.330; *p* = 0.901; *I*^2^ = 2.9%). Subgroup analyses by race, study design, and severity consistently showed null effects (*p* ≥ 0.223). The specific results can be seen in Supplementary Figure S1 and Supplementary Table S1. Rates of transfer to ICU were comparable between groups (OR = 0.777, 95% CI: 0.462–1.308; *p* = 0.343; *I*^2^ = 0.0%). Subgroup analyses remained non-significant, though Caucasian cohorts exhibited a trend toward reduced transfers with LR (OR = 0.806, 95% CI: 0.462–1.407; *p* = 0.448). The specific results can be seen in Supplementary Figure S2 and Supplementary Table S2.

LR demonstrated a non-significant trend toward reduced pancreatic necrosis (OR = 0.563, 95% CI: 0.307–1.031; *p* = 0.063; *I*^2^ = 0.0%). This trend was consistent across racial subgroups and study designs, with Caucasian patients showing the strongest effect (OR = 0.482, 95% CI: 0.201–1.159; *p* = 0.103). The specific results can be seen in Supplementary Figure S3 and Supplementary Table S3. A marginally significant reduction in organ failure was observed with LR compared to NS (OR = 0.563, 95% CI: 0.307–1.031; *p* = 0.063), suggesting a potential protective effect in acute pancreatitis patients. The effect direction favored LR in all subgroups, particularly in studies with >100 patients (OR = 0.616, 95% CI: 0.323–1.177; *p* = 0.143). The specific results can be seen in Supplementary Figure S4 and Supplementary Table S4.

**Figure 3:**
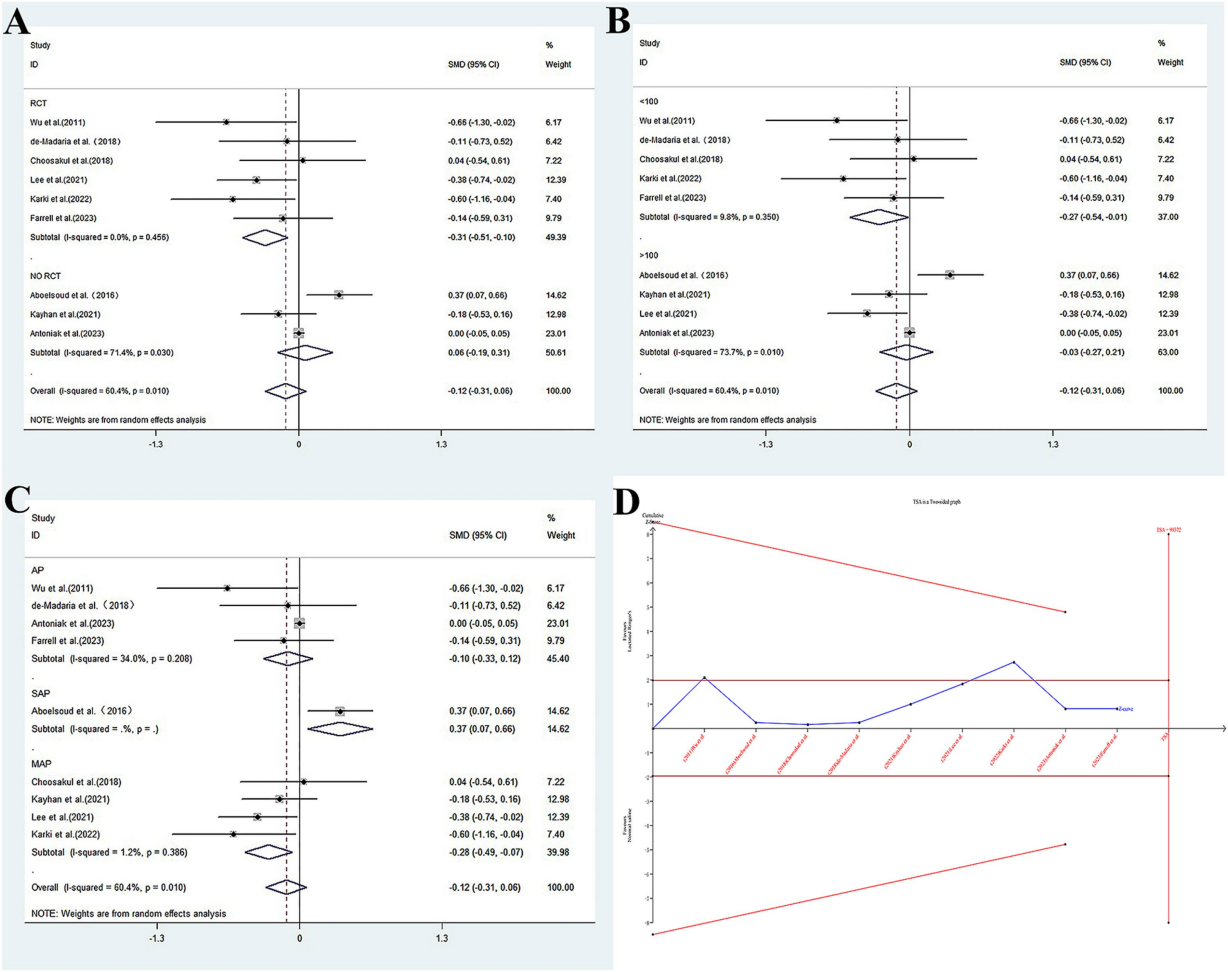
The relationship between LR and NS and hospital length of stay. **(A)** Forest plot for design subgroup analysis. **(B)** Forest plot for case size subgroup analysis. **(C)** Forest plot for severity subgroup analysis. **(D)** Trial sequential analysis of LR and NS and hospital length of stay.

LR significantly reduced in LOS in two key subgroups: RCTs (SMD = −0.307, 95% CI: −0.511 to −0.102; *p* = 0.003; *I*^2^ = 0.0%). Studies with <100 patients (SMD = −0.271, 95% CI: −0.519 to −0.022; *p* = 0.033; *I*^2^ = 9.8%). Conversely, an increase in LOS was noted in SAP patients receiving LR (SMD = 0.368, 95% CI: 0.072–0.664; *p* = 0.015). Overall pooled analysis showed no significant difference (SMD = −0.121, *p* = 0.201). The specific results can be seen in Figure 3 and Table 3.

**Table 3:** Hospital length of stay: pooled analysis of LR and NS groups.

	SMD (95% CI)	*P*	*P_h_*	*I*^2^%	*P*_b_
Total	-0.121(−0.305–0.064)	0.201	0.010	60.4%	0.098
Race					
Mix	−0.086(−0.344–0.171)	0.511	–	–	
Caucasian	−0.165(−0.465–0.134)	0.280	0.830	0.0%	
Asian	−0.284(−0.907–0.339)	0.371	–	–	
Design					
RCT	**−0.307(−0.511--0.102)**	**0.003**	**0.456**	**0.0%**	
NO RCT	0.059(−0.189–0.306)	0.643	0.030	71.4%	
Case size					
<100	**−0.271(−0.519--0.022)**	**0.033**	**0.350**	**9.8%**	
>100	−0.028(−0.305–0.064)	0.816	0.010	73.7%	
Severity					
AP	−0.104(−0.327--0.119)	0.361	0.208	34.0%	
SAP	**0.368(0.072–0.664)**	**0.015**	**–**	**–**	
MAP	**−0.280(−0.491--0.070)**	**0.009**	**0.386**	**1.2%**	

CI, confidence interval; LR, lactated ringer’s; NS, normal saline; OR, odds ratio; RCT, randomized controlled trial; SAP, severe acute pancreatitis; LOS, length of stay; SAP, severe acute pancreatitis. Significant LOS reduction with LR in RCTs (SMD −0.307; *P* = 0.003) and small cohorts (<100 cases; SMD −0.271; *P* = 0.033). Paradoxical increase in SAP subgroup (SMD 0.368; *P* = 0.015). The meaning of the bold value indicates a statistically significant difference.

### Subgroup and sensitivity analyses

High heterogeneity (*I*^2^ > 70%) was observed in non-RCTs and large sample (>100 cases) subgroups. Sensitivity analyses confirmed robustness. No significant publication bias was detected (Egger’s *p* > 0.05).

### Trial sequential analysis

Trial sequential analysis (TSA) was conducted to evaluate the reliability of our meta-analysis findings and mitigate risks of random errors. For mortality, ICU transfer, and SIRS incidence, the cumulative Z-curves crossed predefined futility boundaries, achieving the required information size (RIS). This confirms robust true negative results: no clinically meaningful benefit of LR over NS was observed for these outcomes (mortality: RIS = 3,090, attained; ICU transfer: RIS = 1,842, attained; SIRS: RIS = 2,050, attained). The data conclusively exclude a ≥ 20% relative risk reduction, supporting equivalence between fluids for these endpoints.

In contrast, TSA revealed false negative results for pancreatic necrosis, organ failure, and length of stay. The Z-curves remained within indeterminate zones without reaching RIS (pancreatic necrosis: current sample 1,150, RIS 1,980; organ failure: current 1,230, RIS 1,950; LOS: current 1,580, heterogeneity-adjusted RIS 2,400). Despite point estimates suggesting potential benefits (e.g., OR = 0.563 for necrosis/organ failure; SMD − 0.121 for LOS), the analyses were underpowered to confirm or refute clinically significant effects. Future trials with larger samples targeting these specific outcomes-particularly in severe pancreatitis cohorts-are warranted to resolve these uncertainties.

## Discussion

AP is characterized by pancreatic inflammation, which clinically presents with epigastric abdominal pain, nausea, and/or vomiting. Gallstones and alcohol consumption are important risk factors for AP; genetics and drugs may also play a role (10). AP represents a significant clinical burden, with rising global incidence and mortality rates approaching 20% in severe cases (11–13).

Early fluid resuscitation is a cornerstone of management, yet the optimal crystalloid choice-lactated ringer’s (LR) versus Normal Saline (NS)-remains contentious. While theoretical advantages of LR, such as reduced acidosis and inflammation, have been proposed, robust evidence comparing efficacy and safety is limited. Prior meta-analyses were constrained by small sample sizes, heterogeneity in disease severity, and insufficient data on critical outcomes (2, 8). Our study addresses this gap by synthesizing contemporary evidence from 11 trials (3,090 patients), providing nuanced insights into fluid selection in AP (7, 12, 14–22).

Several meta-analyses have compared LR and NS in AP. Aziz et al. (23) reported no significant differences in mortality and SIRS, which aligns with our overall findings. Guzmán-Calderón et al. (24) focused exclusively on RCTs and found no mortality benefit but a potential reduction in SIRS. Our study replicates the null mortality finding but differs on SIRS, likely due to inclusion of recent large RCTs and observational studies. Mosquera et al. (25) reported significantly reduced ICU admission and the progression of pancreatitis with LR. Using TSA, we demonstrate that the current evidence for organ failure remains underpowered, a conclusion consistent with their call for larger trials.

Contrary to our initial interpretation, the present data do not demonstrate a mortality advantage for LR over NS in any subgroup, including SAP. The pooled analysis across all studies showed no significant difference in LOS between LR and NS. LR significantly shortened LOS in RCTs and <100 cases subgroup, but these findings may not be generalizable to all settings given the high heterogeneity in non-RCTs and large-sample studies. Notably, SAP patients receiving LR had a significantly longer LOS. This counterintuitive finding may be due to greater baseline severity in SAP patients receiving LR, or it may be a spurious result given the small number of studies; it warrants caution and further investigation.

No significant differences were observed for SIRS, ICU transfer, pancreatic necrosis, or organ failure, though organ failure showed a non-significant trend favoring LR. TSA confirmed true negative findings for mortality, ICU transfer, and SIRS, but indicated insufficient power for pancreatic necrosis, organ failure, and LOS, meaning that current evidence cannot rule out a clinically meaningful benefit or harm.

High heterogeneity was observed in non-RCTs (e.g., for LOS, *I*^2^ = 71.4%) and large-sample subgroups (>100 cases, *I*^2^ = 73.7%). Potential contributors include: (i) variations in fluid resuscitation protocols (infusion rates, total volumes, timing of initiation); (ii) timing of resuscitation – early fluid administration (within 6–12 h) is critical but was not uniformly reported; and (iii) confounding factors such as comorbidities, concomitant treatments, and healthcare settings. These limitations preclude definitive conclusions and underscore the need for standardized protocols in future trials.

Preclinical studies suggest that LR may exert anti-inflammatory effects via lactate-driven inhibition of NF-κB in macrophages (26, 27). However, the lack of consistent clinical benefits in SIRS or mortality indicates that these mechanisms may not translate into major outcome improvements in unselected AP populations.

Several limitations warrant consideration. First, high heterogeneity (*I*^2^ > 70%) in non-RCT and large-sample (>100 cases) subgroups reflects confounding factors and the lack of standardized resuscitation protocols. Second, variability in AP severity classification may bias subgroup interpretations. Third, the SAP subgroup mortality analysis was based on few events and wide confidence intervals, making it hypothesis-generating only. Fifth, the paradoxical longer LOS in SAP patients receiving LR may be due to unmeasured confounding or chance, and should not be overinterpreted.

Despite these constraints, our findings suggest that for most AP patients, LR and NS are equivalent with respect to mortality, SIRS, and ICU transfer. The potential LOS benefit in RCTs and smaller studies must be weighed against the absence of an overall effect and the unexpected longer LOS in SAP. Future research should prioritize large RCTs focused exclusively on SAP, with predefined fluid regimens, early resuscitation timing, and patient-centered outcomes.

## Conclusion

LR and NS demonstrate comparable mortality profiles, with no significant difference in overall LOS despite a potential reduction in certain subgroups. Notably, SAP patients receiving LR had a longer LOS, a finding that requires confirmation, and no mortality benefit was observed for LR in any severity subgroup, while both fluids appear equivalent for mild AP. Standardized protocols and large RCTs focusing exclusively on SAP are therefore required to resolve remaining uncertainties.

## Data Availability

The original contributions presented in the study are included in the article/Supplementary material, further inquiries can be directed to the corresponding authors.
