# Supplementary material for: Normal saline vs. lactated ringer’s for fluid resuscitation in acute pancreatitis: a systematic review and meta-analysis
Source: Front Med (Lausanne). 2026 May 8;13:1792711. doi: 10.3389/fmed.2026.1792711 (PMC13195006; doi:10.3389/fmed.2026.1792711)
Supplement: Supplementary file 1 [file Table_1.docx]

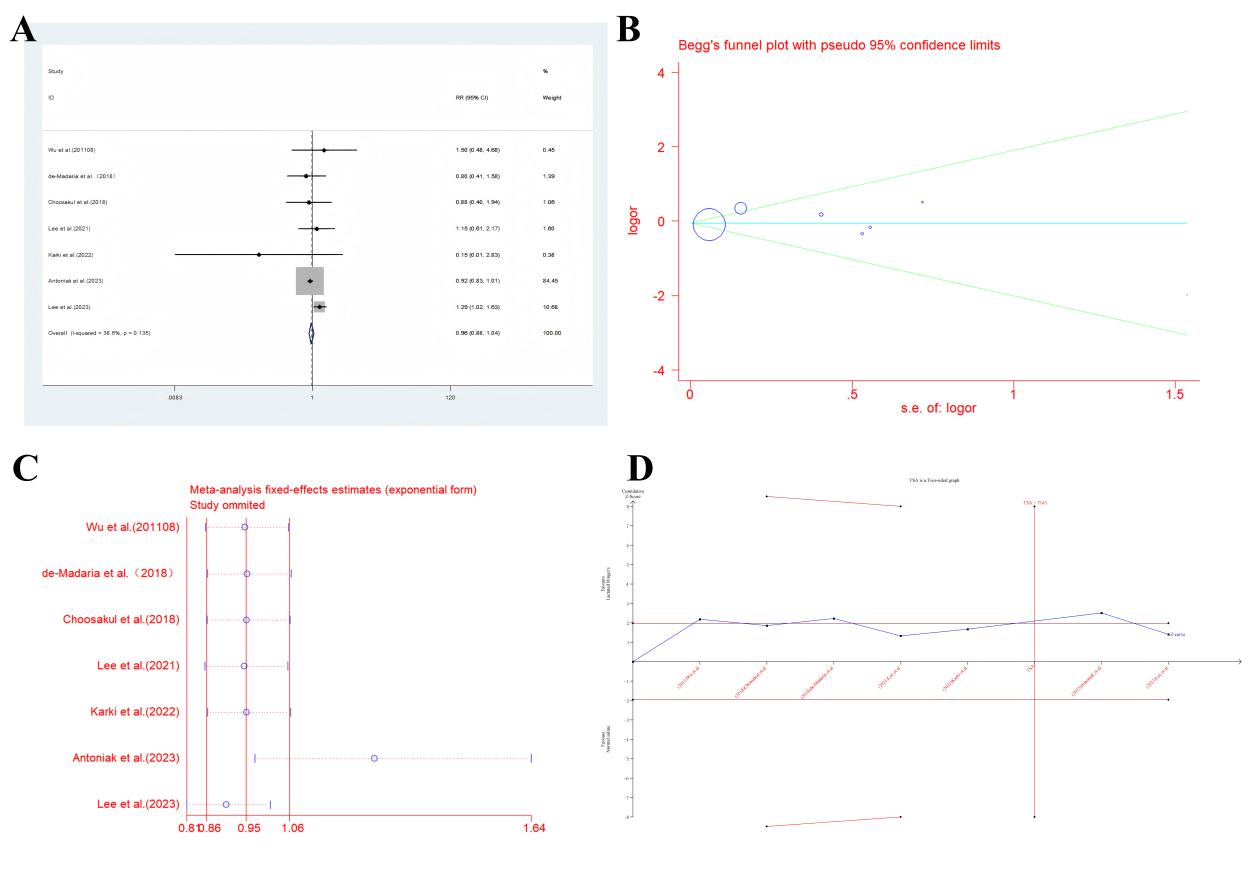


**Supplenmentary Figure S1 The relationship between LR and NS and SIRS.**

**(A)** Forest plot for overall analysis **(B)** The funnel plot for the association between LR and NS and SIRS **(C)** Sensitivity analysis of between LR and NS and SIRS **(D)** Trial sequential analysis of LR and NS and SIRS.

**
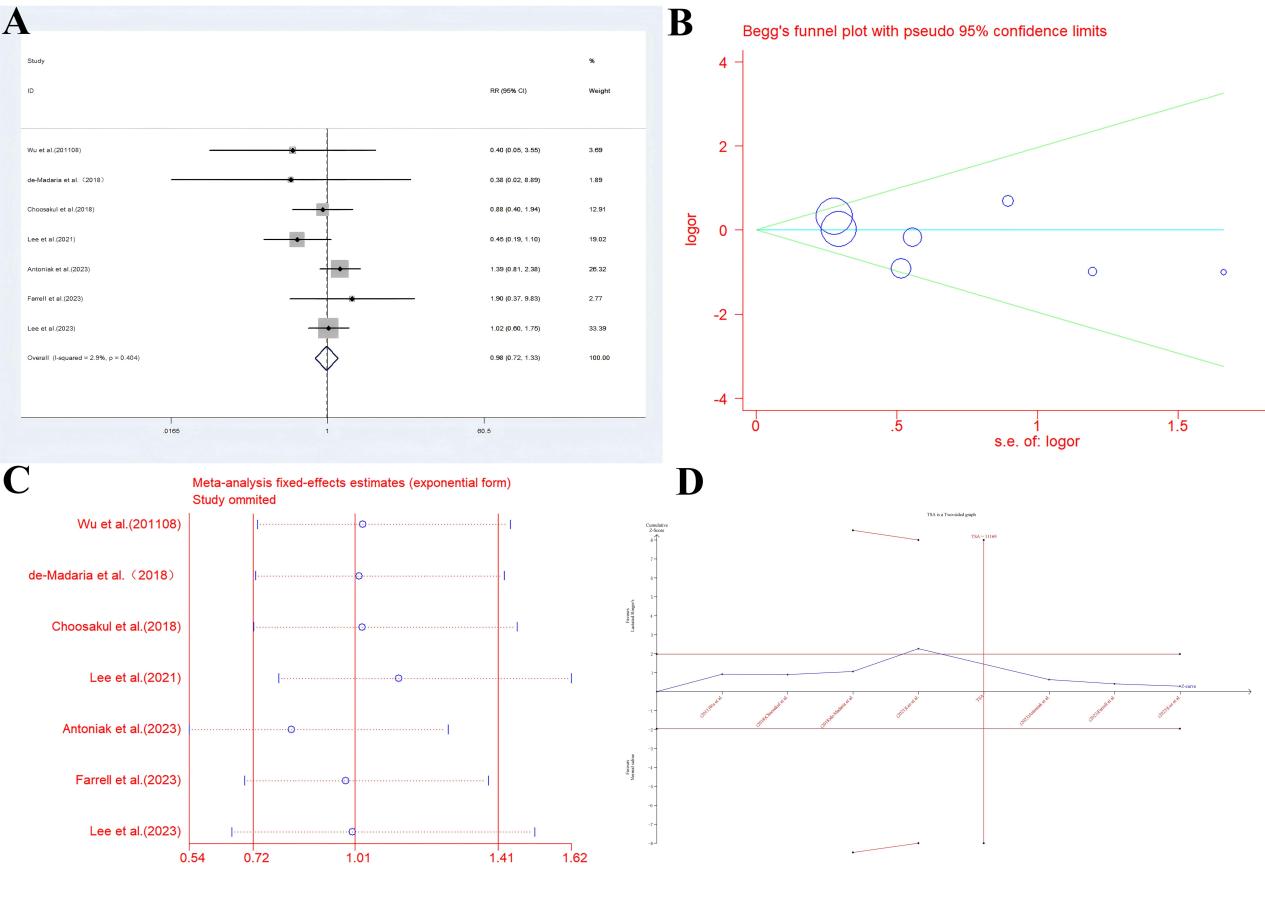
**

**Supplenmentary Figure S2 The relationship between LR and NS and the rates of transfer to ICU. (A)** Forest plot for overall analysis **(B)** The funnel plot for the association between LR and NS and the rates of transfer to ICU **(C)** Sensitivity analysis of between LR and NS and the rates of transfer to ICU **(D)** Trial sequential analysis of LR and NS and the rates of transfer to ICU.

**
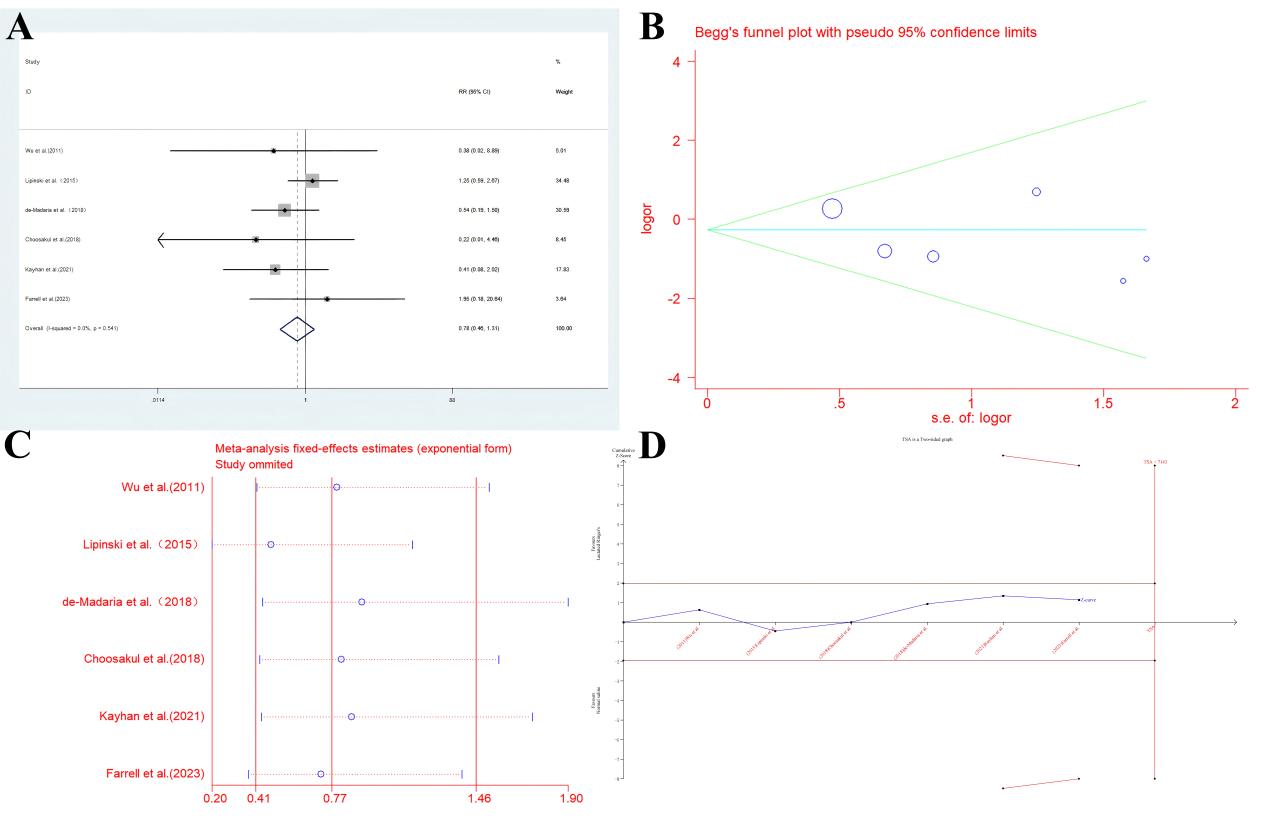
**

**Supplenmentary Figure S3 The relationship between LR and NS and pancreatic necrosis incidence in patients. (A)** Forest plot for overall analysis **(B)** The funnel plot for the association between LR and NS and pancreatic necrosis incidence in patients **(C)** Sensitivity analysis of between LR and NS and pancreatic necrosis incidence in patients **(D)** Trial sequential analysis of LR and NS and pancreatic necrosis incidence in patients.

**
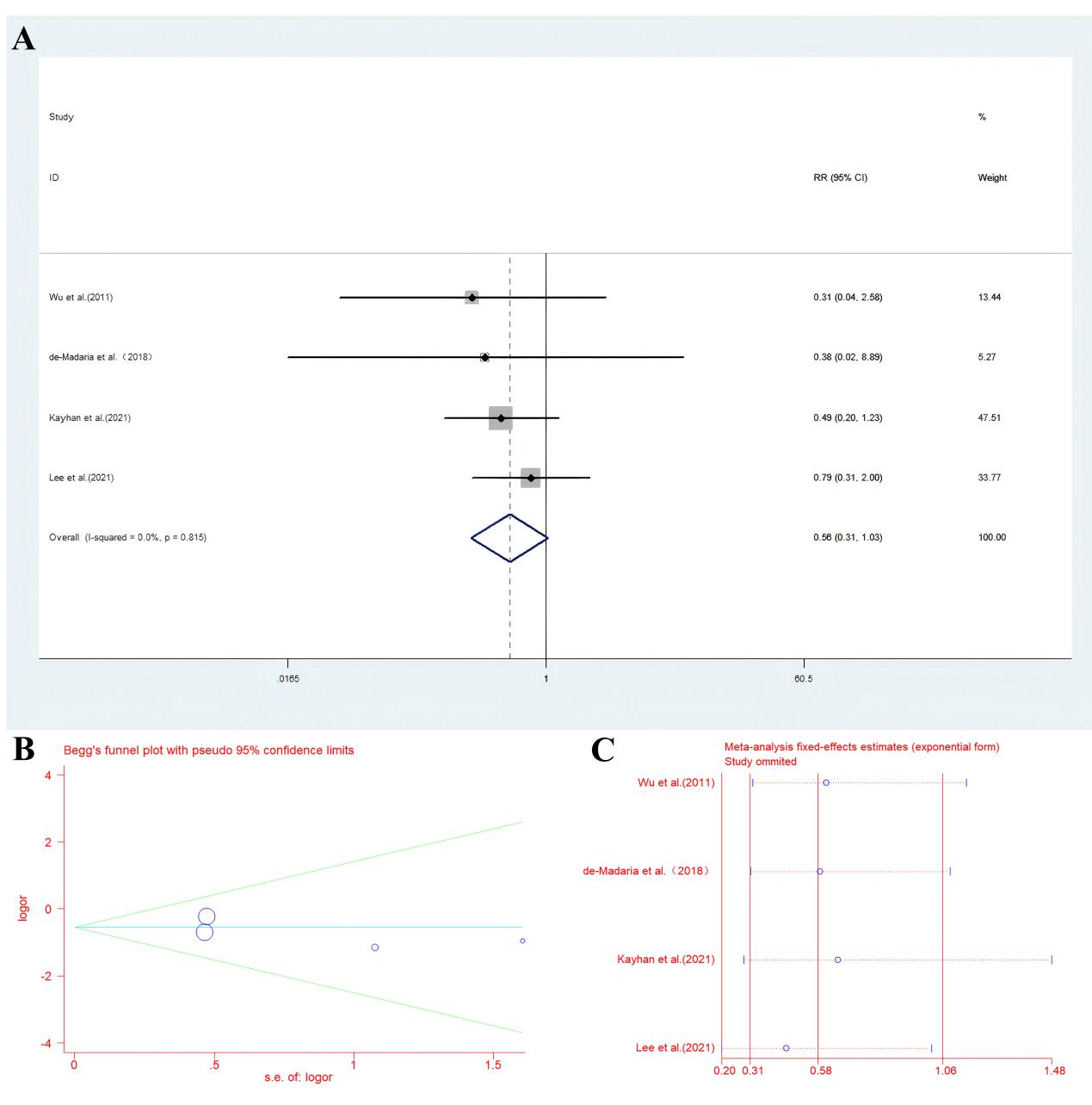
**

**Supplenmentary Figure S The relationship between LR and NS and Organ failure. (A)** Forest plot for overall analysis **(B)** The funnel plot for the association between LR and NS and Organ failure **(C)** Sensitivity analysis of between LR and NS and Organ failure

**Supplenmentary Table S1. Systemic inflammatory response syndrome (SIRS) incidence by resuscitation fluid type (comparative effects of LR and NS)**

|  | **OR (95% CI)** | ***P*** | ***P_h_*** | ***I*^2^%** | ***P*_b_** |
| --- | --- | --- | --- | --- | --- |
| **Total** | 0.958(0.879-1.044) | 0.331 | 0.135 | 38.6% | 0.783 |
| **Race** |  |  |  |  |  |
| Mix | 0.957(0.862-1.063) | 0.411 | 0.050 | 61.6% |  |
| Caucasian | 0.711(0.251-2.015) | 0.520 | - | - |  |
| Asian | 0.612(0.229-1.638) | 0.329 | 0.263 | 20.3% |  |
| **Design** |  |  |  |  |  |
| RCT | 0.948(0.655-1.371) | 0.776 | 0.599 | 0.0% |  |
| NO RCT | 0.950(0.854-1.057) | 0.345 | 0.009 | 85.5% |  |
| **Case size** |  |  |  |  |  |
| <100 | 0.848(0.537-1.339) | 0.478 | 0.509 | 0.0% |  |
| >100 | 0.954(0.859-1.060) | 0.380 | 0.027 | 72.3% |  |
| **Severity** |  |  |  |  |  |
| AP | 0.950(0.855-1.056) | 0.343 | 0.050 | 61.6% |  |
| MAP | 0.932(0.577-1.507) | 0.775 | 0.382 | 0.0% |  |

Abbreviations: CI = confidence interval; LR = Lactated Ringer's; NS = normal saline; OR = odds ratio; RCT = randomized controlled trial; SAP = severe acute pancreatitis.

Note: Random-effects model used for mixed-race and non-RCT subgroups (I² > 50%). No significant intergroup differences observed across subgroups.

**Supplenmentary Table S2. The rates of transfer to ICU following fluid resuscitation with LR or NS**

|  | **OR (95% CI)** | ***P*** | ***P_h_*** | ***I*^2^%** | ***P*_b_** |
| --- | --- | --- | --- | --- | --- |
| **Total** | 0.981(0.723-1.330) | 0.901 | 0.404 | 2.9% | 0.273 |
| **Race** |  |  |  |  |  |
| Mix | 1.010(0.725-1.406) | 0.954 | 0.223 | 29.8% |  |
| Caucasian | 0.383(0.017-8.892) | 0.550 | - | - |  |
| Asian | 0.877(0.397-1.937) | 0.746 | - | - |  |
| **Design** |  |  |  |  |  |
| RCT | 0.681(0.405-1.147) | 0.149 | 0.548 | 0.0% |  |
| NO RCT | 1.183(0.809-1.730) | 0.386 | 0.434 | 0.0% |  |
| **Case size** |  |  |  |  |  |
| <100 | 0.885(0.460-1.700) | 0.713 | 0.655 | 0.0% |  |
| >100 | 1.007(0.694-1.462) | 0.970 | 0.103 | 55.9% |  |
| **Severity** |  |  |  |  |  |
| AP | 1.148(0.801-1.645) | 0.453 | 0.668 | 0.0% |  |
| MAP | 0.625(0.347-1.127) | 0.118 | 0.272 | 17.0% |  |

Abbreviations: CI = confidence interval; LR = Lactated Ringer's; NS = normal saline; OR = odds ratio; RCT = randomized controlled trial; SAP = severe acute pancreatitis.
Note: Non-significant overall effect (OR 0.981; P = 0.901).

**Supplenmentary Table S3. Pancreatic necrosis incidence in patients receiving LR vs. NS**

|  | **OR (95% CI)** | ***P*** | ***P_h_*** | ***I*^2^%** | ***P*_b_** |
| --- | --- | --- | --- | --- | --- |
| **Total** | 0.777(0.462-1.308) | 0.343 | 0.541 | 0.0% | 0.326 |
| **Race** |  |  |  |  |  |
| Mix | 1.042(0.181-5.998) | 0.963 | 0.417 | 0.0% |  |
| Caucasian | 0.806(0.462-1.407) | 0.448 | 0.276 | 22.3% |  |
| Asian | 0.225(0.011-4.457) | 0.328 | - | - |  |
| **Design** |  |  |  |  |  |
| RCT | 0.575(0.249-1.326) | 0.194 | 0.685 | 0.0% |  |
| NO RCT | 0.956(0.403-1.372) | 0.912 | 0.212 | 35.9% |  |
| **Case size** |  |  |  |  |  |
| <100 | 0.575(0.249-1.326) | 0.194 | 0.685 | 0.0% |  |
| >100 | 0.956(0.432-2.115) | 0.912 | 0.212 | 35.9% |  |
| **Severity** |  |  |  |  |  |
| AP | 0.650(0.270-1.563) | 0.336 | 0.586 | 0.0% |  |
| SAP | 1.250(0.585-2.670) | 0.565 | - | - |  |
| MAP | 0.348(0.085-1.421) | 0.141 | 0.732 | 0.0% |  |

Abbreviations: CI = confidence interval; LR = Lactated Ringer's; NS = normal saline; OR = odds ratio; RCT = randomized controlled trial; SAP = severe acute pancreatitis.

Note: Non-significant overall effect (OR 0.777; P = 0.343). Caucasian subgroup showed non-significant trend toward reduced transfers with LR (OR 0.806; P = 0.448).

**Supplenmentary Table S4. Organ failure outcomes stratified by fluid type**

|  | **OR (95% CI)** | ***P*** | ***P_h_*** | ***I*^2^%** | ***P*_b_** |
| --- | --- | --- | --- | --- | --- |
| **Total** | *0.563(0.307-1.031)* | *0.063* | 0.815 | 0.0% | 0.406 |
| **Race** |  |  |  |  |  |
| Mix | 0.654(0.282-1.514) | 0.321 | 0.428 | 0.0% |  |
| Caucasian | 0.482(0.201-1.159) | 0.103 | 0.880 | 0.0% |  |
| **Design** |  |  |  |  |  |
| RCT | 0.626(0.278-1.409) | 0.258 | 0.688 | 0.0% |  |
| NO RCT | 0.493(0.198-1.229) | 0.129 | - | - |  |
| **Case size** |  |  |  |  |  |
| <100 | 0.332(0.058-1.915) | 0.218 | 0.916 | 0.0% |  |
| >100 | 0.616(0.323-1.177) | 0.143 | 0.479 | 0.0% |  |
| **Severity** |  |  |  |  |  |
| AP | 0.332(0.058-1.915) | 0.218 | 0.916 | 0.0% |  |
| MAP | 0.616(0.323-1.177) | 0.143 | 0.479 | 0.0% |  |

Abbreviations: CI = confidence interval; LR = Lactated Ringer's; NS = normal saline; OR = odds ratio; RCT = randomized controlled trial; SAP = severe acute pancreatitis.

**Note:** Marginally significant reduction with LR (OR 0.563; P = 0.063). Consistent direction of effect across subgroups, strongest in Caucasians (OR 0.482; P = 0.103). Benefit most pronounced in studies with >100 patients (OR 0.616; P = 0.143).
